# Overexpression of 9-*cis*-Epoxycarotenoid Dioxygenase Cisgene in Grapevine Increases Drought Tolerance and Results in Pleiotropic Effects

**DOI:** 10.3389/fpls.2018.00970

**Published:** 2018-08-03

**Authors:** Rongrong He, Yuan Zhuang, Yumeng Cai, Cecilia B. Agüero, Shaoli Liu, Jiao Wu, Shuhan Deng, Michael A. Walker, Jiang Lu, Yali Zhang

**Affiliations:** ^1^Department of Viticulture and Enology, College of Food Science and Nutritional Engineering, China Agricultural University, Beijing, China; ^2^Department of Viticulture & Enology, University of California, Davis, Davis, CA, United States; ^3^Center for Viticulture and Enology, School of Agriculture and Biology, Shanghai Jiao Tong University, Shanghai, China

**Keywords:** abscisic acid, 9-*cis*-epoxycarotenoid dioxygenase, grapevine, embryogenic culture, seed dormancy, transformation

## Abstract

9-*cis*-epoxycarotenoid dioxygenase (NCED) is a key enzyme involved in the biosynthesis of abscisic acid (ABA), which is associated with drought tolerance in plants. An osmotic-inducible *VaNCED1* gene was isolated from a drought-resistant cultivar of *Vitis amurensis* and constitutively overexpressed in a drought-sensitive cultivar of *Vitis vinifera*. Transgenic plants showed significantly improved drought tolerance, including a higher growth rate and better drought resistant under drought conditions, compared to those of wild-type (WT) plants. After water was withheld for 50 days, the upper leaves of transgenic plants remained green, whereas most leaves of WT plants turned yellow and fell. Besides the increase in ABA content, overexpression of *VaNCED1* induced the production of jasmonic acid (JA) and accumulation of JA biosynthesis-related genes, including *allene oxide cyclase* (*AOC*) and *12-oxophytodienoate reductase* (*OPR3*). Moreover, transgenic plants possessed advantageous physiological indices, including lower leaf stomatal density, lower photosynthesis rate, and lower accumulation of proline and superoxide dismutase (SOD), compared to those of WT plants, indicating increased resistance to drought stress. Quantitative real time polymerase chain reaction (RT-qPCR) analysis revealed that overexpression of *VaNCED1* enhanced the expression of drought-responsive genes, such as *ABA-responsive element* *1* (*ABRE1*)*, ABRE binding factors 2* (*ABF2*), *plasma membrane intrinsic proteins 2* (*PIP2*), *C-repeat/DRE-Binding Factor 4* (*VvCBF4*) and *ABA-insensitive 5* (*ABI5*). Although the development of transgenic plants was delayed by 4 months than WT plants, because of seed dormancy and abnormal seedlings, the surviving transgenic plants provided a solid method for protection of woody plants from drought stress.

## Introduction

Water is essential for plant growth; it carries nutrients to support plant growth and serves as a medium for *in vivo* reactions. However, climate change in recent years has accelerated the expansion of drylands, threatening the growth, yield, and quality of plants because of drought stress. Faced with the challenge of climate and environmental changes, breeders need to add new traits to improve tolerance to abiotic stresses (Chapman et al., 2012). Although conventional breeding has increased the commercial quality of most modern crops, genetic methods also provide powerful tools to accelerate the progress of plant breeding.

Abscisic acid (ABA) is an important plant hormone involved in various physiological processes in plants, such as the response to abiotic stresses (Seo and Koshiba, 2002; Fujita et al., 2011; Awan et al., 2017). When plants are subjected to abiotic stress, ABA rapidly accumulates, inducing stomatal closure to reduce water loss *via* transpiration (Zhang et al., 2008; Estrada-Melo et al., 2015).

Abscisic acid is synthesized from C40-carotenoids, which are oxidatively cleaved from neoxanthin by 9-*cis*-epoxycarotenoid dioxygenase (NCED) to yield xanthoxin, the direct C15 precursor of ABA (Schwartz et al., 1997). NCED is considered the rate-limiting enzyme in ABA biosynthesis (Kende and Zeevaart, 1997; Koornneef et al., 1998; Rodrigo et al., 2006), and this gene was first characterized from the ABA-deficient maize mutant *viviparous-14* (*vp14*) (Tan et al., 1997). Subsequently, *NCED* genes have been identified in other plants, such as tomato (*Lycopersicon esculentum*) (Burbidge et al., 1999), bean (*Phaseolus* *vulgaris*) (Qin and Zeevaart, 1999), cowpea (*Vigna unguiculata*) (Iuchi et al., 2000), avocado (*Persea* *americana*) (Chernys and Zeevaart, 2000), *Arabidopsis* (Iuchi et al., 2001), *Vitis vinifera* (Soar et al., 2004), peanut (*Arachis hypogaea*) (Wan and Li, 2005), and orange (*Citrus sinensis*) (Rodrigo et al., 2006). *VvNCED1* and *VvNCED2* were first characterized from *Vitis vinifera* L. ‘Shiraz’ genomic DNA by Soar et al. (2004). *VvNCED* gene expression has been shown to be induced by water deficit (Qin and Zeevaart, 1999; Chernys and Zeevaart, 2000; Wan and Li, 2005; Rodrigo et al., 2006) and salt stress (Iuchi et al., 2000).

Abscisic acid-deficient plants, *aba1*, *aba2*, and *aba3* were generated from *Arabidopsis* (Koornneef et al., 1982, 1984); *notabilis* (*not*) (Burbidge et al., 1999; Thompson et al., 2004) and *sitiens* (Aroca et al., 2008) were generated from tomato; and *vp14* was generated from maize. These ABA-deficient plants showed reduced plant growth both under well-watered and drought stress conditions. Additionally, overexpression of *NCED* gene or exogenous ABA application greatly improved the growth of these ABA-deficient plants (Thompson et al., 2004; Aroca et al., 2008). Overexpression of *NCED* gene resulted in ABA accumulation and increased drought tolerance in tomato (Thompson et al., 2000), cowpea (Iuchi et al., 2001), tobacco (Qin and Zeevaart, 2002; Pedrosa et al., 2017), peanut (Wan and Li, 2006), rice (Sultana et al., 2014), petunia (Estrada-Melo et al., 2015), cotton (Souza et al., 2016), and *Arabidopsis* (Tong et al., 2017); moreover, it improved salt tolerance in creeping bent grass (Aswath et al., 2005). Recently, a citrus *CsNCED3-*transformed tobacco exhibited an increased ABA content and drought resistance (Pedrosa et al., 2017). Overexpression of *NCED* in tomato led to negative pleiotropic effects, such as overguttation, leaf-margin chlorosis, and seed dormancy (Thompson et al., 2000). To avoid these negative effects, different inducible promoters were introduced to replace the constitutively expressed promoter 35S, such as stress-inducible promoter *rd29A* (Estrada-Melo et al., 2015), Super-promoter (consisting of a trimer of the octopine synthase upstream activating element linked to the mannopine synthase promoter) (Thompson et al., 2000), dexamethasone (DEX)-inducible promoter (Qin and Zeevaart, 2002), and the AtNCED3p promoter (*AtNCED3* gene promoter) (Wan and Li, 2006). Although *NCED* has been transformed in many species, it has not been transformed in woody plants yet.

‘Zuoshan-1’ (ZSY) is a grapevine variety breeding from Chinese local grapevine *Vitis amurensis* species, which has great performance on drought tolerance (Qu and Deng, 1994). In our study, a drought stress-induced *VaNCED1* gene from ZSY was transformed into a drought-sensitive grapevine variety *V. vinifera* L. ‘Thompson Seedless’ (TS), which is a very popular table grape variety in the world. We hypothesized that overexpression of *VaNCED1* would increase ABA content and improve drought tolerance of grapevines.

## Materials and Methods

### Plant Materials and Growth Condition

ZSY and TS were collected from Shangzhuang agricultural experimental station (China Agricultural University, Beijing, China). The tissue culture was developed from buds, which were induced from shoots of grapevine. Pre-embryogenic callus of TS was developed from anther culture grown on PIV medium (Franks et al., 1998) and subcultured on NB medium (Le Gall et al., 1994).

Tissue culture was propagated on WP medium (Lloyd and McCown, 1981) (PhytoTechnology Laboratories, Lenexa, KN, United States), containing 3% sucrose and 0.6% agar (pH 5.7 ± 0.05) in a growth room under 16 h 24°C/8 h 16°C light/dark cycle and 50% relative humidity.

Plants with 10 fully expanded leaves at a height of approximately 80 cm grown in 30 × 30 × 20 cm pot in soil were selected for drought treatment. The growth conditions were as follows: room temperature (25°C), and light intensity = 400 μmol⋅m^−2^⋅s^−1^. Water was withheld in the growth room for 50 days for dehydration treatment. Control plants were supplied with deionized water every 2 days. Plant growth was measured every 2 days after dehydration until day 6, proline content and superoxide dismutase (SOD) activity were measured in plant leaves after dehydration for 6 days, and photosynthesis rate, transpiration rate, and water use efficiency (WUE) were measured in plant leaves after dehydration for 26 days. In each of these assays, 3–5 plants repeats were used.

### Polyethylene Glycol Treatments and Transcript Analysis of *NCED*

The response of ZSY and TS to osmotic stress was tested. Plants with five fully expanded leaves in tissue culture were treated with liquid MS (Murashige and Skoog, 1962) (PhytoTechnology Laboratories, Overland Park, KS, United States) containing 10% polyethylene glycol (PEG) 6000 and 20% PEG6000 separately. The grapevine leaves were collected at 0, 4, 8, 24, 48, 96, and 192 h after treatment. The primers for *Vitis NCED* (forward: 5′-CACACGCCGCCCTATACTTC-3′; reverse: 5′-CACCATACCTCTGCTCTCCA-3′) were used to determine the expression levels of *NCED* in ZSY and TS. The normalized expression level of *NCED* in each cultivar at 0 h was used as a control value (expression set to 1).

### Total RNA Isolation and Quantitative Real-Time PCR (RT-qPCR)

Total RNA was extracted from harvested samples using a Quick RNA isolation kit (Huayueyang, Beijing, China). First strand cDNA was synthesized from DNase-treated (Promega, Madison, WI, United States) total RNA using ImProm-II TM reverse transcriptase (Promega, Madison, WI, United States). Five hundred nano gram of total RNA was used in a 10-μL reaction mixture. The reactions were performed using a Roto-Gene Q real-time polymerase chain reaction (PCR) machine (Qiagen, Hilden, Germany) in a 10-μL reaction mixture containing 5 μL of SYBR Green Supermix, 0.2 μL of 10 μM primers, 50 ng of cDNA, and ddH_2_O added to a total volume of 10 μL.

*Vitis EFα* (XM_002284888) (forward primer: 5′-TCCAAGGCAAGGTACGATG-3′; reverse primer: 5′-CAGAGATGGGGACAAATGG-3′) and *Vitis actin* (AF369524.1) (Pastenes et al., 2014) (forward primer: 5′-AGCTGGAAACTGCAAAGAGCAG-3′; reverse primer: 5′-ACAACGGAATCTCTCAGCTCCA-3′) were used as two reference genes for data normalization. Experiments were carried out using Power SYBR Green PCR Master Mix (Applied Biosystems, Warrington, United Kingdom) in a StepOne^TM^ real-time PCR system (Applied Biosystems). The thermal cycling conditions were as follows: 95°C for 10 min, followed by 40 cycles of 95°C for 10 s, 58°C for 15 s, and 72°C for 30 s. Specificity of the individual RT-qPCR amplifications was assessed using heat dissociation curves from 55 to 95°C after the final cycle. The fold change in mRNA expression was estimated using threshold cycles by the 2^−ΔΔCT^ method (Livak and Schmittgen, 2001). Primers for RT-qPCR were designed using Beacon Designer ver. 7.0 (Premier Biosoft, Palo Alto, CA, United States).

### *VaNCED1* Cloning, Sequencing, and Gene Structure Analysis

The genomic DNA of ZSY was isolated from the stem using the cetyltrimethyl ammonium bromide (CTAB) method (Murray and Thompson, 1980). A pair of primers to amplify the full-length open reading frame (ORF) of *VaNCED1* was designed using DNAman 6.0 software (Lynnon Biosoft LLC., San Ramon, CA, United States)^1^. The forward primer, UTRF, was 5′-CATCACACTACCCAACAGCC-3′, and the reverse primer, UTRR, was 5′-TCCTCGTCCTTTACACTCTCG-3′. PCR was performed in a 50-μL reaction system containing 50 ng of genomic DNA, 1 μM of each primer, 200 mM of deoxynucleotide triphosphates (dNTPs), 5 μL of Pfu DNA polymerase 10× reaction buffer with MgSO_4_, and 1.25 U Pfu DNA polymerase (Promega, Beijing, China). The PCR thermal cycling conditions were as follows: 95°C for 2 min, followed by 35 cycles of 95°C for 1 min, 58°C for 30 s, and 72°C for 2 min, and extension at 72°C for 5 min. PCR products were then isolated from the agarose gel blocks by electrophoresis and purified using QIAquick gel extraction kit (Qiagen, Valencia, CA, United States). The purified DNA was then cloned into the pGEM T-Easy vector (Promega, Beijing, China) and sequenced. The ORFs in the amplified DNA sequence were identified using the ORF finder at NCBI ^2^.

The resulting PCR products were sequenced and translated to obtain an amino acid sequence. VaNCED1 protein was aligned with NCED from *V. vinifera* ‘Pinot Noir’ (GenBank accession no. VV78X205727.5). *VaNCED1* and other reported *NCED* sequences were compared using DNAman 6.0 software. The full-length NCED amino acid sequences of other species were downloaded from NCBI.

### Plasmid Constructs

The specific primers, NCEDbam (5′-ATAGGATCCATGGCTTCTCCTGC-3′) and NCEDsal (5′-TATGAGCTCTCAAGCTTGCTTCTC-3′) were used to amplify the full-length ORF of *VaNCED1* using Pfu DNA polymerase (Promega, Madison, WI, United States) from the VaNCED-T easy vector, and then it was introduced into the binary vector pBI121 (Clontech Labs, Palo Alto, CA, United States) using *BamH*I and *Sal*I restriction enzymes (New England Biolabs, Ipswich, MA, United States). In the new construct, the BamHI-SalI fragment replaced the *gus* reporter gene and *nos* terminator gene (Jefferson, 1987). This new construct was named pBI121-VaNCED-nos. The specific primers NOSecoF (5′-TATGAATTCGTCGACGAGCTCGAATTTCCCC-3′) and NOSecoR (5′-AGTGAATTCCCGATCTAGTAACATAGATGA-3′) were used to amplify the *nos* sequence from the pBI121 plasmid, which was digested using the *EcoR*I restriction enzyme (New England Biolabs, Ipswich, MA, United States). After digestion, the *nos* fragment was introduced into the pBI121-VaNCED-nos construct, and the direction of *nos* was confirmed by PCR using the primers, NCEDbamF (5′-ATAGGATCCATGGCTTCTCCTGC-3′) and NOSecoR (5′-TATGAGCTCTCAAGCTTGCTTCTC-3′). This binary vector was transformed into *Escherichia coli* and verified by PCR. This new construct was named pBI121-VaNCED; it contained the *nptII* gene driven by the *nos* promoter and the *VaNCED1* gene driven by the *CaMV* *35S* promoter, and both were stopped by the *nos* gene. The vector was introduced into the disarmed *Agrobacterium* *tumefaciens* strain EHA105 (Hood et al., 1993) using the electroporation method.

### Transformation and Identification of *VaNCED*

*Agrobacterium tumefaciens* was cultured overnight at 28°C in liquid Luria-Bertani (LB) medium (10 g/L peptone, 5 g/L yeast extract, and 5 g/L sodium chloride) containing 25 mg/L kanamycin. The cells were collected by centrifugation at 5000 × *g* for 3 min. The pellets were then resuspended to approximately 1 × 10^8^ cells/mL in liquid MS medium supplemented with 20 μM acetosyringone (PhytoTechnology Laboratories, Overland Park, KS, United States). The resuspended *Agrobacterium tumefaciens* cells were dropped onto the embryogenic callus of TS (Agüero et al., 2006). The callus was cultured on PT medium lacking activated charcoal and supplemented with 4 μM picloram, 2.3 μM thidiazuron (TDZ), and 100 μM acetosyringone for 48 h. The callus was then divided into small clusters and subcultured on PT medium containing 100 μg/mL kanamycin and 300 μg/mL cefotaxime. After selection, the germinated embryos were transferred to WP medium. The plantlets developed from embryos were then transferred to the growth room, and cultured in 0.5-L pots with a potting mixture consisting of vermiculite, sand, and soil (1:1:1, v/v/v).

Genomic DNA was isolated from the young leaves of WT and putative transgenic plants according to a modified CTAB method. PCR detection of *nos* gene was carried out with specific forward primer (nos-F: 5′-ATTGCGGGACTCTAATCATA-3′) and reverse primer (nos-R: 5′-ATCGTTCAAACATTTGGCA-3′). PCR amplifications were carried out at 94°C for 5 min, followed by 30 cycles of 94°C for 30 s, 60°C for 30 s, and 72°C for 60 s, with a final elongation at 72°C for 10 min. PCR products were separated by 1% (w/v) agarose gel electrophoresis.

### Analysis of Phytohormones and Transcript of Phytohormone-Related Genes

Abscisic acid, jasmonic acid (JA), and salicylic acid (SA) were extracted as described by Liu et al. (2016). The fourth and fifth fully expanded leaves from WT and transgenic plants were ground to powder in liquid nitrogen for hormone extraction. Then, 150 mg of the ground powder was transferred to 2-mL tubes containing 50 μL of the internal standard working solution and 0.5 mL extraction solvent (2-propanol/H_2_O/concentrated HCl = 2/1/0.002, v/v/v), which were then shaken for 30 min at 4°C. The extraction from the suspension was carried out with 1 mL of dichloromethane by shaking at 4°C for 30 min. The mixture was centrifuged at 10,000 × *g* for 5 min. The lower phase was transferred and concentrated. The concentrated residue was then redissolved in 100 mL of methanol. For high-performance liquid chromatography-electrospray ionization tandem mass spectrometry (HPLC-ESI-MS/MS) analysis, 50 mL of the sample solution was injected into a reverse-phase C18 column (see **Table 1** for HPLC gradient).

**Table 1:** High-performance liquid chromatography (HPLC) gradient program used to separate phytohormones.

Time (min)	Methanol (%)	0.01% Formic acid (%)	Flow rate (mL/min)
10	5.0	95.0	0.15
40	15.0	85.0	0.15
50	85.0	15.0	0.15
60	5.0	95.0	0.15
70	5.0	95.0	0.15

The expression of *allene oxide cyclase* (*AOC*) (GenBank accession no. GSVIVT01036445001), *12-oxophytodienoate reductase* *3* (*OPR3*) (GenBank accession no. NM_001281046.1), *phenylalanine ammonia lyase* (*PAL*) (Locus name: GSVIVG01025703001) and *isochorismate synthase* (*ICS*) (Locus name: GSVIVT01008052001) in WT and transgenic plants under normal conditions and after withholding water for 30 days was determined by RT-qPCR as described above. The primers for these genes were as follows: *VvAOC* forward (5′-CGCTCCACTCCACACACTAC-3′) and reverse (5′-CTTCCACGGTCTCTCTCATT-3′); *VvOPR3* forward (5′-AGGCGTGGAAGAAGGTTGTG-3′) and reverse (5′-GCTGGTTGATGATATGGGTG-3′), *VvPAL* forward (5′-AATGGTTGGTGATGTGTTG-3′) and reverse (5′-ATGAGTCTGTTCCGTTCC-3′); and *VvICS* forward (5′-GTTCTTCCGACATATTCA-3′) and reverse (5′-CATTCAGATGATACATTAGC-3′). To analyze total *NCED* expression, the forward primer TNF (5′-CACACGCCGCCCTATACTTC-3′) and the reverse primer TNR (5′-CACCATACCTCTGCTCTCCA-3′) were designed. To amplify the *VaNCED1* cisgene, a forward primer VANF (5′-AGAAGCAAGCTTGAGAAT-3′) at the junction region between the *EcoR*I restriction site and the *VaNCED1* sequence, and a reverse primer VANR (5′-TATTTTGTTTTCTATCGCGT-3′) at the second *EcoR*I enzyme site were designed.

### Dehydration Response Evaluation

Dehydration and growth assays were performed using regenerated plants growing in 30 × 30 × 20 cm pots in the growth room. WT and transgenic plants with 10 fully expanded leaves at a height of approximately 80 cm were subjected to drought stress by withholding water for 50 days. Three-to-five plants were used in this assay. Plant height was measured at 10:30 in the morning every 2 days until there was no shoot elongation.

### Stomatal Bioassays

The fourth and fifth fully expended leaves were sampled to determine stomatal density, epidermal cell density, stomatal aperture, and stomatal size. Each sample was immersed in 20% NaClO solution overnight, stained with safranin for 4 min, and then washed twice with distilled water. The leaf was placed on a glass slide gently then put a cover glass on it. The slides were photographed with a Jenoptik ProgRes C5 camera attached to a Carl Zeiss Scope A1 microscope and analyzed using ProgRes^®^ CapturePro 2.8.8 software (Jenoptik Optical Systems, Jena, Germany). For statistical analysis of cell density, stomatal aperture, and stomatal size, three leaves were sampled for each of three plants, and 250–300 microscopic fields were examined for each plant. Stomatal density = number of stomata in field/field area (μm^2^). Epidermal cell density = number of epidermal cells in field/ field area (μm^2^).

### Photosynthesis (P), Transpiration (T) Rates, and Conductance (S)

Photosynthesis (P), transpiration (T) rates, and stomatal conductance (S) were measured using a portable photosynthesis system LI-COR LI-6400 (Li-Cor, Lincoln, NE, United States) in the morning (10:00 to 11:00 AM). The first fully expanded leaves were selected for the assay. WUE was defined as the P/T ratio and derived from the measured *P* and *T* values. These parameters were measured under normal conditions and 26 days after withholding water. Five measurements were performed for each plant, and three plants were tested for each line.

### Proline Measurement

Proline content was measured using a ninhydrin-based colorimetric assay (Bates et al., 1973). The fourth and fifth fully expanded leaves from WT and transgenic plants were sampled on days 0 and 6 after dehydration. Proline concentration was determined from a standard concentration curve.

### SOD Activity Assay

Superoxide dismutase (SOD, EC 1.15.1.1) activity was measured using SOD assay kit (Nanjing Jiancheng Bio-Institute, Nanjing, China). The fourth and fifth fully expanded leaves were sampled on days 0 and 6 after dehydration. Tissue samples (0.5 g) were homogenized in 4 mL of a reaction buffer containing 1% (w/v) polyvinylpyrrolidone and 50 mM sodium phosphate buffer (pH = 7). The homogenate was centrifuged at 10,000 × *g* for 30 min at 4°C. The resulting supernatant was collected as a crude enzyme extract, and SOD activity was determined (Parida et al., 2004).

### Transcript Analysis of Drought-Related Genes

The expression of *ABA-responsive element binding protein 1* (*AREB1*) (Zandkarimi et al., 2015), *ABRE binding factors 2* (*ABF2*) (GenBank accession no. FQ380275.1), *Plasma membrane intrinsic proteins 2* (*PIP2*) (GenBank accession no. KJ697715.1), *C-repeat/DRE-Binding Factor 4* (*VvCBF4*) (Zandkarimi et al., 2015) and *ABA-insensitive 5* (*ABI5*) (GenBank accession no. XM_010655778.2) in WT and transgenic plants under normal conditions and after withholding water for 30 days was analyzed by RT-qPCR as described above. The primers for these genes were as follows: *VvAREB1* forward (5′-CTTCCATATACTCCTTGACC-3′) and reverse (5′-AGGCAATGTCAAAGAACCC-3′); *VvABF2* forward (5′- GCCATGACTCTCTCTCCTGT-3′) and reverse (5′-GAACCTTCTACCTCCAACTA-3′); *VvPIP2* forward (5′-GTTTGGGGGCTGCTGTTATC-3′) and reverse (5′-GGTAGAAGGCTGCAATGGCT-3′), *VvCBF4* forward (5′-ACCCTCACCCGCTCGTATG-3′) and reverse (5′-CCGCGTCTCCCGAAACTT-3′); and *ABI5* forward (5′-GCCATGACTCTCTCTCCTGT-3′) and reverse (5′-GAACCTTCTACCTCCAACTA-3′).

### Data Analysis

All experimental data were expressed as the means of at least three independent biological repeat and each repeat has three replicates. The data were compared using Duncan’s multiple range test. A *P*-value < 0.05 was considered statistically significant. All statistical analyses were performed using IBM SPSS Statistics V22.0 (SPSS, Chicago, IL, United States).

## Results

### Changes in the Expression of *NCED* After PEG Treatment

**FIGURE 1:**
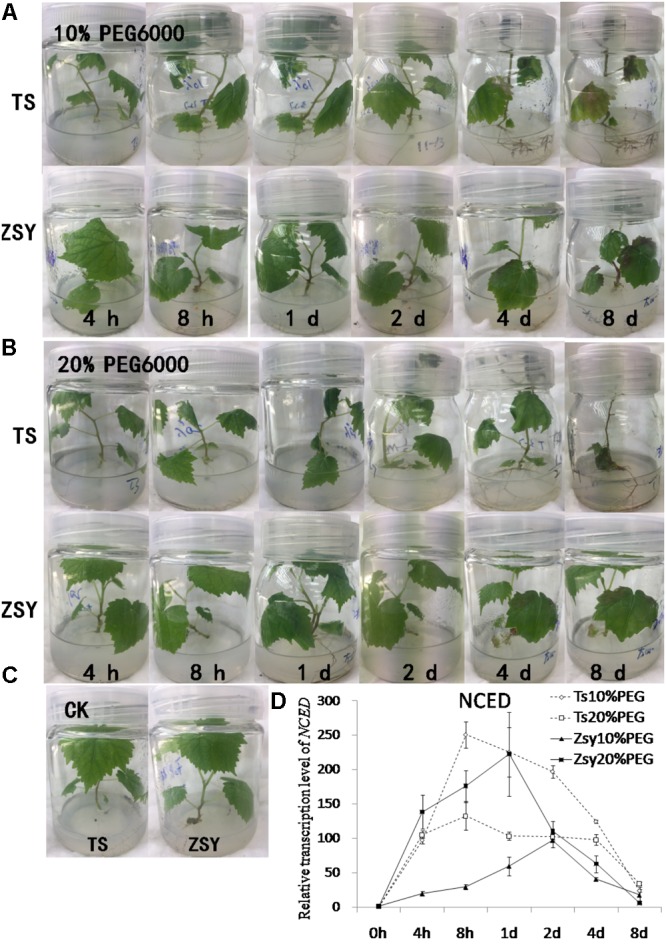
Phenotypes of ‘Thompson seedless’ (TS) and ‘Zuoshan-1’ (ZSY) plants under different stress conditions *in vitro*. The osmotic stress treatments were examined at 0, 4, 8 h, 1, 2, 4, and 8 days in the growth room. Fully expanded leaves of 3–4 plants were treated with 10% PEG6000 to induce mild osmotic stress **(A)**, 20% PEG6000 to induce severe osmotic stress **(B)**, or liquid MS for control conditions **(C)**. Determination of the relative transcript levels of *NCED* in ‘Zuoshan-1’ and ‘Thompson Seedless’ in response to osmotic stress treatment **(D)**. The leaves were obtained for the total RNA isolation. *EFα* and *Vitis actin* were used as two reference genes for data normalization. Data are expressed as the mean ± SD from 3 independent experiments.

To compare osmotic tolerance of ZSY and TS, the plants were subjected to tissue culture medium containing 10 or 20% PEG6000 (**Figure 1**). ZSY showed better resistance than TS. Wilting was not observed in ZSY subjected to 10% PEG6000 treatment, whereas TS showed wilting after 4 h, followed by severe wilting, chlorosis, and water loss symptoms (**Figure 1A**). At the highest concentration of PEG6000 (20%), TS plants showed wilting at 4 h, and ZSY showed mild water loss at 8 h and wilt symptoms on day 1 (**Figure 1B**). Plants added to the same volume of liquid MS were used as a control (**Figure 1C**). PEG6000 treatment (10%) upregulated the expression level of *NCED* in ZSY and reached the peak on day 2. The expression level of *NCED* in TS was much higher than that in ZSY, and peaked earlier at 8 h (**Figure 1D**). In TS, the expression level of *NCED* under 20% PEG6000 treatment was significantly lower than that under 10% PEG6000, while in ZSY, the expression level of *NCED* under 20% PEG6000 treatment was higher than that under 10% PEG6000 treatment. The difference in *NCED* expression between the two plants indicated that TS, but not ZSY, could not respond to stress induced by 20% PEG6000. These two experiments showed that TS was much more sensitive to water stress than ZSY, and *NCED* expression responded to water stress in both ZSY and TS under low concentration of PEG6000. Therefore, we deduced *NCED* might play an important role in water stress, and the regulatory ability of *NCED* in ZSY could maintain its drought-tolerant phenotype under severe dehydration.

### Identification of Grapevine *NCED* Gene

**FIGURE 2:**
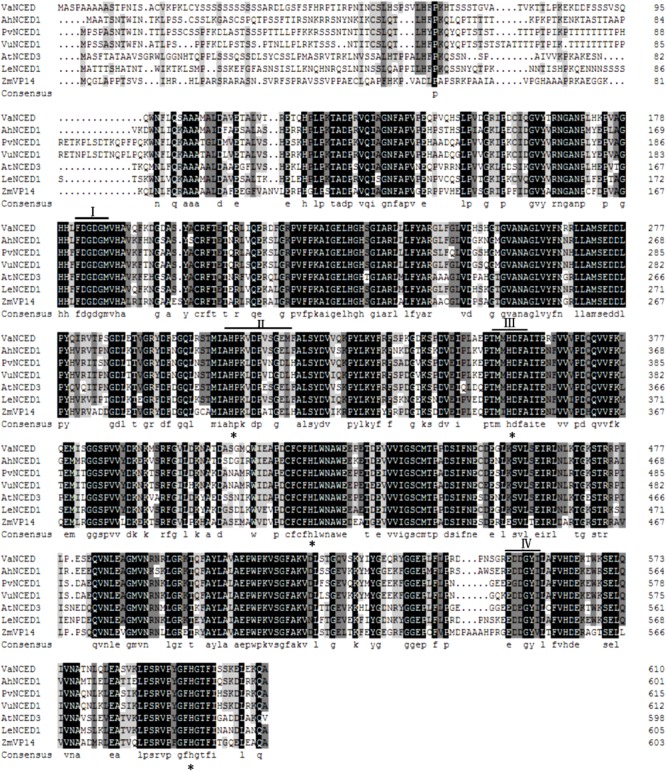
Multiple alignments of the deduced amino acids of VaNCED1 with other NCEDs from bean PvNCED1 (AF190462), cowpea VuNCED1 (AB030293), peanut AhNCED1 (AJ574819), tomato LeNCED1 (Z97215), *Arabidopsis* AtNCED3 (AY056255), and maize ZmVP14 (U95953). Black color represents the homologous regions. I, II, III, and IV indicate the conserved sequences. The four histidine residues are marked with asterisks.

The full-length DNA of *VaNCED1* consisted of 1833 bp nucleotides. *VaNCED1* contained an ORF encoding a polypeptide of 611 amino acids (GenBank accession number MG603069), with a calculated molecular weight for the putative protein of 67.193 kDa and an isoelectric point of 7.84. The amino acid sequence of VaNCED1 shared 97% identity with that of NCED1 (AM468138.1) from *V. vinifera* ‘Pinot Noir’ (**Supplementary Figure S1**), 97% with NCED1 (NM_001281270) from *V. vinifera* ‘Tannatand,’ 97% with NCED1 (AY337613.1) from *V. vinifera* ‘Shiraz,’ and 78% with that of NCED2 (NM_001281271.1). The four different amino acids were not located within the four conserved areas. VaNCED1 shared a highly conserved core region with orthologous proteins from various plant species (**Figure 2**). The protein BLAST results showed that the predicted protein sequence of VaNCED1 shared 71.21, 71.65, 71.86, 71.97, 72.64, and 75% identity with NCEDs from *P.* *vulgaris*, *Zea* *mays vp14*, *Solanum lycopersicum, V.* *unguiculata*, *Arabidopsis* *thaliana*, and *Arachis* *hypogaea.* This high similarity suggested that VaNCED1 is a member of the NCED family. Conserved regions and four conserved histidine residues around these regions (Burbidge et al., 1997; Tan et al., 1997) were all found in VaNCED. Analysis of sequence similarity suggested that *VaNCED1* encoded a putative NCED.

### Transformation and Screening of Transgenic Grapevines

**FIGURE 3:**
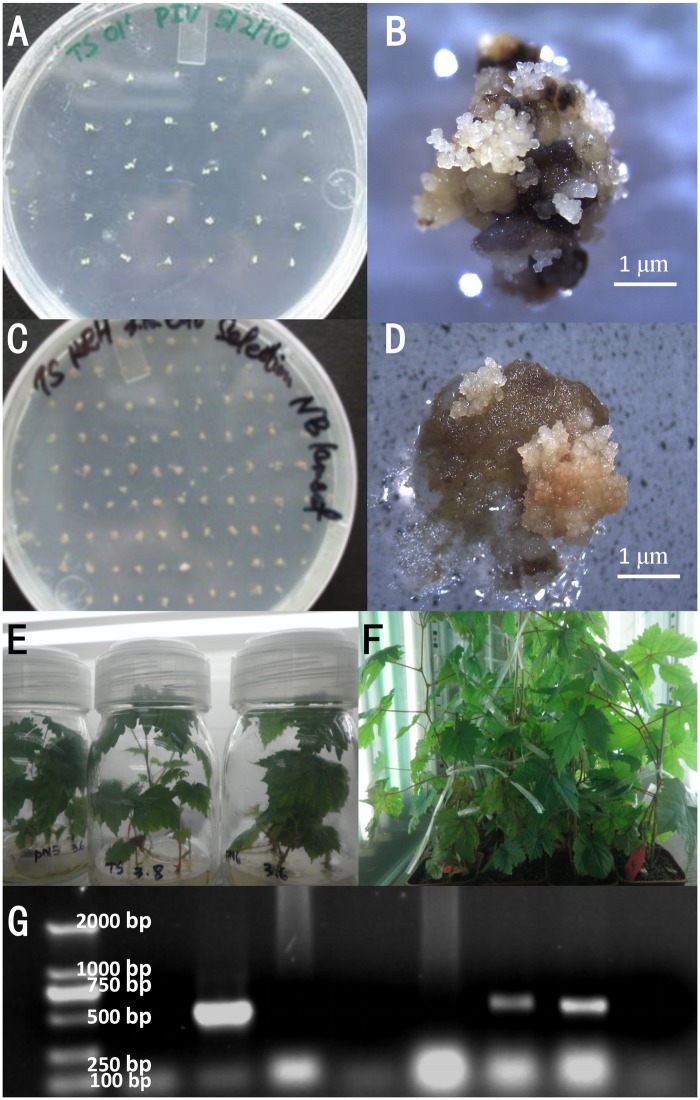
Transformation and selection of plants. Separated anthers were inoculated on PIV medium **(A)**. Induced somatic embryogenic culture from anthers after cultivation **(B)**. Callus of Thompson seedless on the selection medium for 2 months after transformation with the *VaNCED1* gene **(C)** and enlarged view **(D)**. Regenerated wild-type (WT) and putative transgenic plants *in vitro* **(E)** and in soil **(F)**. Validation of putative transgenic plants by PCR amplification of the *nos* terminator **(G)**. M, DL2000 marker; WT, wild-type negative control; P, plasmid pBI121-VaNCED1; 1–6, putative transgenic grapevine lines. Bar = 1 mm.

Anthers were separated under a microscope and inoculated on PIV medium (**Figure 3A**). Pre-embryogenic calli with a yellowish color and compact structure were successfully induced on PIV medium in 2 months (**Figure 3B**) and were then subcultured on NB medium for proliferation.

The plasmid pBI121-VaNCED1 harboring *VaNCED1* was constructed and transformed into TS. After 2 months of selection, the pre-embryogenic callus shrank and turned brown on the selection medium (**Figure 3C**). The putative transgenic callus was distinguished by its white color and rapid growth rate (**Figure 3D**). The germination of transgenic somatic embryos took 3–4 months longer than that of the WT embryos. Few somatic embryos developed into plants (**Supplementary Figure S2A**), whereas most somatic embryos transformed into malformed plants without normal cotyledon and root formation (**Supplementary Figure S2B**). Six transgenic callus clusters were selected from 800 clusters on the selection medium, and six constitutively overexpressed *VaNCED1* (CVAN) putative lines were successfully regenerated from these six embryogenic transformants (**Figure 3E**). The plants were then transplanted into pots in a growth room (**Figure 3F**). Two lines were confirmed *nos* gene-positive by PCR detection (**Figure 3G**).

### Changes in the Leaf Phytohormones and Phytohormone-Related Genes in Transformants

**FIGURE 4:**
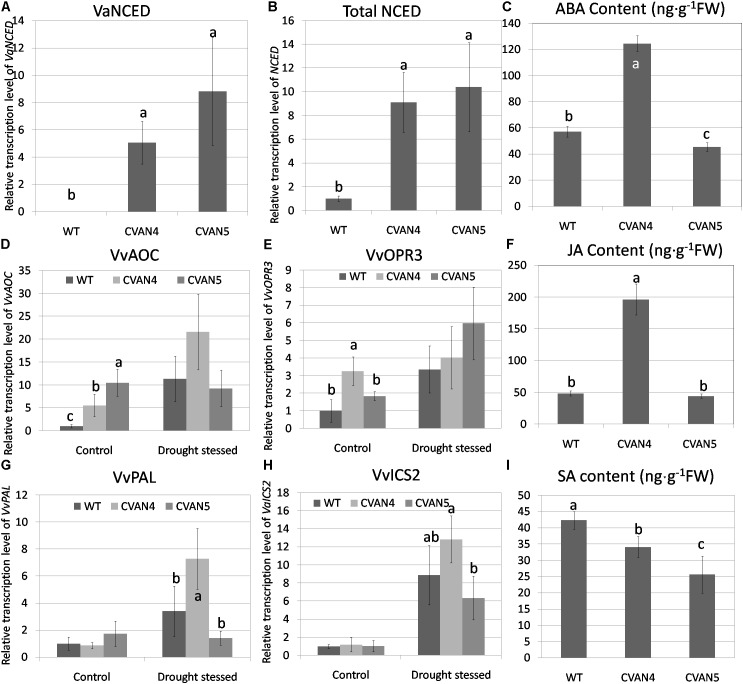
Determination of phytohormone and relative transcript levels of phytohormone-related genes. *VaNCED1* mRNA content **(A)**, total *NCED* mRNA content **(B)**, abscisic acid (ABA) content (ng/g) **(C)**, *VvAOC* mRNA content **(D)**, *OPR3* mRNA content **(E)**, JA content (ng/g) **(F)**, *VvPAL* mRNA content **(G)**, *VvICS* mRNA content **(H)**, and SA content (ng/g) **(I)** of the WT and transgenic lines under normal conditions. The leaves were obtained for the total RNA isolation. *EFα* and *Vitis actin* were used as two reference genes for data normalization. Data are expressed as the mean ± SD from 3 independent experiments. Different letters indicate significant differences (*P* < 0.05) determined by Duncan’s multiple range test (*P* < 0.05) using SPSS statistical software. Gene expression in the WT plants under the same conditions was defined as 1. Because *VaNCED1* cannot be detected in WT, The expression level of *VaNCED1* in transgenic plants are calculated by defined total *NCED* in WT as 1.

To measure the expression of exogenous *VaNCED1*, primers were designed according to the specific region in the restriction enzyme site of the plasmid pBI121-VaNCED. *VaNCED1* expression was not detected in the WT plants; the expression level of *VaNCED1* in transgenic plants are calculated by defined total *NCED* in WT as 1. CVAN4 and CVAN5 showed a 5.06- and 8.82-fold accumulation of *VaNCED1* transcripts (**Figures 4A,B**). We then determined the expression level of total *NCED* mRNA and ABA in the WT and transgenic lines. Primers were designed according to the conserved coding region of *VaNCED1* and *VvNCED1*, and mRNA was amplified from both endogenous *VvNCED1* and exogenous *VaNCED1*. The expression of total *NCED* in CVAN4 and CVAN5 lines was 9.1- and 10.39-fold higher than that in the WT plants (set as 1) respectively under control conditions (**Figure 4B**). ABA content in CVAN4 was significantly higher than that in WT and CVAN5 plants, with a 2.18-fold increase under control conditions (**Figure 4C**), whereas ABA content in CVAN5 slightly decreased, compared to that in the WT plants.

Besides ABA, JA has diverse roles under abiotic stresses and is associated with ABA biosynthesis. *AOC* and *OPR3* are two important genes in JA biosynthesis (Schaller and Stintzi, 2009). In our study, both *VvAOC* and *VvOPR3* expression levels increased in WT plants after dehydration treatment (11.32-fold and 3.36-fold respectively compared to control condition). These two genes in transgenic lines were all increased after dehydration than normal condition (**Figures 4D,E**). JA content in CVAN4 and CVAN5 plants were 4.06- and 0.91-fold of that present in the WT plants under control conditions, which was consistent with ABA content (**Figure 4F**).

Salicylic acid was reported to be involved in physiological and metabolic responses in plants (Hayat et al., 2010). PAL and ICS are two enzymes associated with SA synthesis (Chen et al., 2009). The expression level of *VvPAL* and *VvICS* showed no significant difference under control conditions (**Figures 4G,H**). However, the expression level of *VvPAL* and *VvICS* showed no difference between the transgenic and WT lines. However, these two genes all respond to drought treatment. The content of SA in the transgenic lines decreased than that in the WT lines.

### *VaNCED1* Improved Growth Rate and Drought Stress Response of Grapevine

**FIGURE 5:**
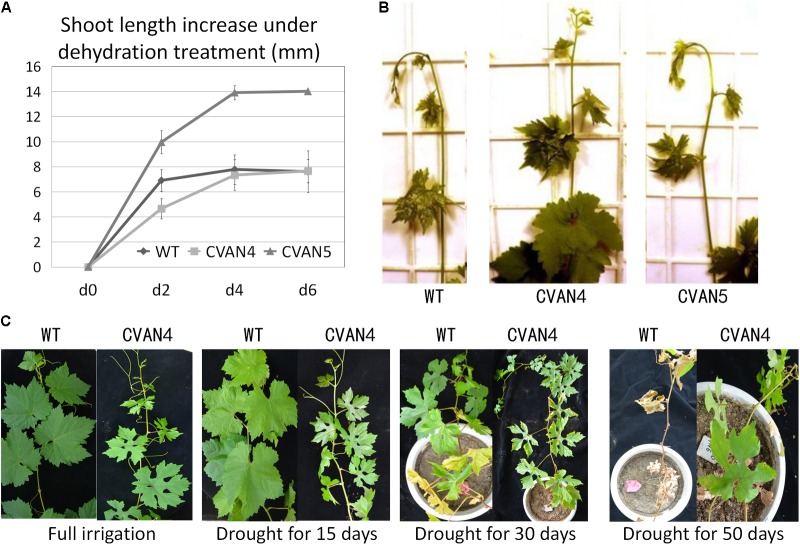
Improved plant growth rate and drought resistance. Increased growth rate of transgenic and non-transgenic plants after dehydration treatment **(A)**. Plant length was measured at 0, 2, 4, and 6 days. Values are means ± SD for three independent experiments. Shoot tips of the WT and transgenic plants after dehydration for 6 days **(B)**. Drought tolerance of WT and transgenic plants (CVAN4) in a growth room **(C)**. Images were obtained for plants without irrigation on days 0, 15, 30, and 50.

The transgenic plants exhibited faster growth and better drought tolerance, compared to the WT plants under drought condition (**Figure 5**). Shoot length was measured on days 0, 2, 4, and 6. Under normal conditions, transgenic CVAN5 plants (7.9 cm) grew faster than CVAN4 (5.1 cm) and WT (5.25 cm) plants (**Figure 5A**). After 4 days without watering, the shoot length significantly increased in the CVAN4 (2.68 cm) and CVAN5 (3.95 cm) transgenic plants, compared to that in the WT plants (0.875 cm). After dehydration for 6 days, the shoot tip of the WT plants showed severe wilting symptoms (−0.18 cm), whereas CVAN5 plants showed slight wilting (0.1 cm), and CVAN4 plants showed no stress symptoms (0.32 cm) (**Figure 5B**). These results indicated that under normal conditions, the CVAN5 plants grew faster than the CVAN4 and WT plants, whereas under drought conditions, the CVAN4 plants showed better drought resistance.

There were no visible phenotypic differences between the WT and transgenic plants when fully irrigated (**Figure 5C**). On day 15 of dehydration, the leaves of the WT and CVAN4 plants showed dryness symptoms. After withholding water for 30 days, only the margins of leaves in CVAN4 plants turned yellow and showed wilting, whereas approximately half of full leaves turned yellow in the WT plants. After 50 days of drought treatment, most leaves of the WT plants turned yellow and fell; however, the upper leaves of *VaNCED1* transgenic plants were still green. The transgenic plants in this experiment exhibited improved drought tolerance.

### *VaNCED1* Altered Stomatal Density and Photosynthesis Rate of Grapevine

**FIGURE 6:**
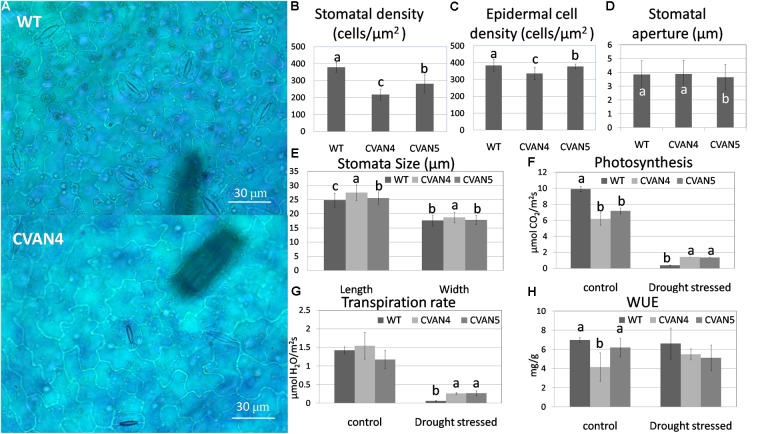
Epidermal images of the WT and transgenic (CVAN4) plants under × 400 magnification **(A)**. Stomatal **(B)** and epidermal cell **(C)** density, stomatal aperture **(D)**, and stomatal size **(E)** of the WT and transgenic plants. Values represent the means ± SD (*n* = 250–300). Photosynthesis rate **(F)**, transpiration rate **(G)**, and water use efficiency **(H)** under normal conditions and after dehydration for 26 days. Values are means ± SD for three independent experiments. Values with the same letter are not significantly different compared to the WT according to Duncan’s multiple range test (*P* < 0.05). Bar = 30 μm.

To further analyze drought tolerance, we measured the surface leaf cells and photosynthesis. The average stomatal density of the CVAN4 and CVAN5 plants was 42.54 and 25.77% less than that of the WT plants (**Figures 6A,B**). The density of epidermal cells in the CVAN4 and CVAN5 plants was 12.23 and 1.54% lower than that in the WT plants (**Figure 6C**). The stomatal aperture in CVAN5 plants was less than that in the WT and CVAN4 plants (**Figure 6D**). Moreover, CVAN4 plants exhibited slightly larger stomata with an average size of 27.51 μm (length) by 18.71 μm (width) μm, whereas the size of the CVAN5 and WT plants was 25.55 by 17.83 μm and 24.79 by 17.67 μm, respectively (**Figure 6E**). The reduced stomatal density suggested decreased CO_2_ exchange, which was consistent with the lower rate of photosynthesis in transgenic lines, compared to that in WT plants under normal conditions (**Figure 6F**). However, a higher rate of photosynthesis under dehydration conditions implied that transgenic lines exhibited enhanced drought resistance, compared to the WT line. Similarly, the transpiration rates of transgenic plants were also significantly higher than those of the WT plants after treatment (**Figure 6G**). WUE decreased in the transgenic plants under normal and drought conditions (**Figure 6H**).

### Effects of *VaNCED1* on Proline and SOD Contents

**FIGURE 7:**
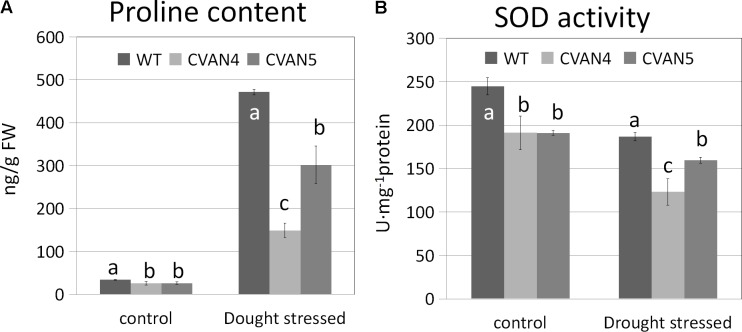
Proline **(A)** and SOD **(B)** contents in the WT and transgenic plants on days 0 and 6 under dehydration treatment. Values are means ± SD for three independent experiments. Values with the same letter are not significantly different compared to the WT under the same condition according to Duncan’s multiple range tests (*P* < 0.05).

Accumulation of inorganic ions and compatible solutes is a mechanism of osmotic adjustment in plants (Ashraf and Bashir, 2003). Proline is a very important compatible solute, which can reduce the osmotic potential of plant tissue. Accumulation of proline in plants improves their resistance to drought stress (Yamada et al., 2005). SOD is an important component of the antioxidant defense system, which counteracts reactive oxygen species (ROS)-induced toxicity (Van Camp et al., 1990). We determined proline and SOD contents in the WT and transgenic plants under normal conditions and after 6 days of drought treatment. Accompanying the increased drought tolerance, the proline content (**Figure 7A**) and level of the antioxidant enzymes SOD (**Figure 7B**) was lower in the transgenic lines compared with the WT plants both under normal and drought stress conditions. Since proline and SOD levels are indicative of damage in plants, this suggested that transgenic plants were less sensitive to drought stress than WT plants.

### Transcript Analysis of Stress-Responsive Genes

**FIGURE 8:**
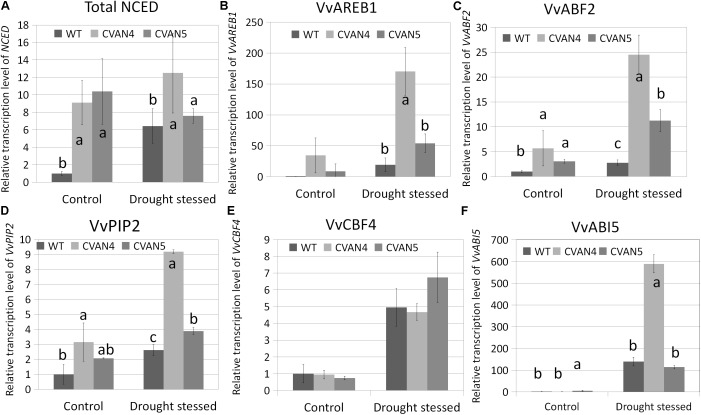
Determination of relative transcript levels of drought-related genes. Total *NCED* expression **(A)**, *VvAREB*1 **(B)**, *VvABF*2 **(C)**, *VvPIP2* **(D)**, and *VvCBF5* **(E)** mRNA expression in the WT and transgenic lines under normal conditions and after dehydration for 30 days. The leaves were obtained for the total RNA isolation. *EFα* and *Vitis actin* were used as two reference genes for data normalization. Data are expressed as the mean ± SD from 3 independent experiments. Different letters indicate significant differences (*P* < 0.05) determined by Duncan’s multiple range test (*P* < 0.05) using SPSS statistical software. Gene expression in the WT plants under the same conditions was defined as 1.

Besides stomatal closure, plants respond to increased ABA concentration by inducing the expression of dehydration-related genes to help plants survive under stress conditions (Urano et al., 2009; Cutler et al., 2010). These genes encode proteins that help plants to maintain water uptake under drought stress by accumulating solutes in the cytoplasm, thus lowering water potential, preserving cell turgor, and minimizing water loss. Therefore, we compared the expression of stress-responsive genes between the WT and transgenic plants under normal conditions and after 30 days of drought stress. First, we measured the expression of total *NCED* in the WT and transgenic plants under drought stress. Compared with the WT plants under normal conditions, the expression level of total *NCED* in the WT plants under drought stress showed a 6.42-fold increase, whereas total *NCED* expression in the CVAN4 and CVAN5 lines changed by 1.37- and 0.73-fold, respectively (**Figure 8A**). ABRE-binding protein (AREB)/ABRE binding factors (ABFs) binds to the ABA-responsive element (ABRE) in their promoter region (Fujita et al., 2005). *AREB*/*ABFs* are involved in ABA and stress signaling in *Arabidopsis* (Uno et al., 2000; Fujita et al., 2005), rice (Hossain et al., 2010), and grapevine (Boneh et al., 2012; Zandkarimi et al., 2015). PIPs represent one of four groups of aquaporins present in the plasma and vascular membranes that play important roles in plants under drought stress (Aroca et al., 2006). As shown in **Figure 8**, *VvAREB1* (**Figure 8B**), *VvABF2* (**Figure 8C**), and *VvPIP2* (**Figure 8D**) were upregulated in the transgenic lines under both normal and dehydrated conditions, which was consistent with the increased drought resistance. The expression level of *VvCBF4* showed no difference under control condition, while the expression level of CVAN5 increased more than the WT and CVAN4 (**Figure 8E**). Compared to the expression level of *VvABI5* in WT, the level of *VvABI5* increased in CVAN4 (1.24-fold) and CVAN5 (5.45-fold) (**Figure 8F**). And the expression level of *VvABI5* was drastically induced by drought stress, the increasing fold is 139.86 (WT), 473.81 (CVAN4) and 20.94 (CVAN5). These results showed that *VaNCED1* gene could greatly improve drought tolerance in plants.

## Discussion

‘Zuoshan-1’ is a variety selected from native grapevine in China, with excellent biotic and abiotic stress resistance. We chose this genotype as a donor with a pool of candidate resistant genes in this study. First, we compared water stress resistance and expression level of the candidate gene *NCED* in ZSY with those in a drought-sensitive grapevine TS. Based on the phenotype and RT-qPCR assay results, we concluded that *NCED* was induced by water stress in ZSY (**Figure 3**), and this gene contributed to its drought-tolerant phenotype. We then cloned *NCED* from ZSY and compared this gene with *NCED*s from other species. The sequence showed a high similarity with drought stress-induced NCEDs, which suggested that VaNCED encoded a putative NCED (**Figure 1**). VaNCED1 shared the highest similarity with AhNCED1 (75%) from peanut, which has been shown to be upregulated by drought stress (Wan and Li, 2005). Additionally, overexpression of *AhNCED1* was found to improve drought stress tolerance in transgenic *Arabidopsis* (Wan and Li, 2006). Thus, we suggested that *NCED1* in ZSY might be a good candidate gene for increasing plant drought tolerance.

### *VaNCED1* Induced Seed Dormancy of Somatic Embryos

The first distinguishable feature of the *VaNCED1* transformants was the arrestment of somatic embryos and the lower transformation rate. The germination of transgenic somatic embryos exceeded 6 months, which was 3–4 months longer than that required for the WT somatic embryos; additionally, only few embryos were induced well into plants. A similar finding has also been reported in transgenic *Arabidopsis* with the chimeric gene *pABRE:NCED* (Nonogaki et al., 2014). The transgenic seeds of *Arabidopsis* exhibited deep dormancy, which lasted more than 3 months longer than that of the control seeds.

It is well known that ABA metabolism is highly regulated when plants are grown in a water-deficient environment. Moreover, ABA plays a central role in the induction and maintenance of seed dormancy; additionally, it inhibits the transition from embryonic to germination growth (Rodríguez-Gacio et al., 2009). The earliest ABA-deficient mutants are germinating revertants selected from non-germinating GA-deficient mutants (Koornneef et al., 1982). Inhibition of NCED may shorten the germination time of tomato and lettuce (Awan et al., 2017). Although low concentrations of ABA (0–1 μM) stimulate the elongation of embryos and normal embryo induction, high concentrations of ABA (10–100 μM) result in growth inhibition of somatic embryos. This phenomenon also has been reported in cactus *Copiapoa tenuissima* Ritt (Lema-Rumińska et al., 2013) and transgenic tomato with the *LeNCED* gene (Tung et al., 2008). These studies can help to explain the arrestment of somatic embryos in *VaNCED1*-overexpressing grapevines in this study.

The overexpression of *VaNCED1* also increased the malform rate of somatic embryos. In this study, the selected putative transgenic somatic embryogenic lines (**Figure 3D**) were less vigorous and darker than those observed in our previous thaumatin-like protein transgenic experiment (He et al., 2017) previously. The cotyledonary somatic embryos shown in **Supplementary Figure S2B** represent plants developed with two separated cotyledons, but without a normal shoot apical meristem. Similar effect of ABA was found after ABA (5 × 10^−7^ M) treatment on somatic embryos of *Liriodendron tulipifera.* The induction of somatic embryos was inhibited, meanwhile malformed somatic embryos with separated cotyledons were developed, but they rarely transfered into plantlets (Merkle et al., 1990).

Although the arrestment of somatic embryo developmentis an important negative effect of *NCED* overexpression, the most popular propagation method for grapevine is cuttage, the use of which can prevent this drawback. Other visual phenotypes associated with *NCED* gene in transgenic tomato, such as leaf-flooding, guttation, and yellow interveinal sectoring (Thompson et al., 2000; Tung et al., 2008) were not observed in the transgenic grapevine plants.

### *VaNCED1* Altered Phytohormone Content and Gene Expression

To investigate the relationship between the transgene *VaNCED1* and endogenous *VvNCED1*, we determined the expression level of *VaNCED1* and total *NCED* (**Figures 4**, **8**). Our results indicated that the expression of exogenous *VaNCED1* increased the expression of total *NCED* expression in transgenic plants under normal condition (**Figure 8A**). This suggested the presence of a positive feedback mechanism of *NCED* gene expression in grapevine. This feedback mechanism was also reported in *Arabidopsis* (Nonogaki et al., 2014).

Up-regulation of total *NCED* expression in the transgenic plants also contributed to ABA accumulation in CVAN4 plants. Unexpectedly, ABA content in CVAN5 plants was lower than that in the WT plants, opposite to the results of total *NCED* expression (**Figures 4A–C**). This might explained by the complex transportation mechanism of ABA. Although ABA is synthesized from both roots and shoots, the content of ABA in each organ depends on a complex transportation mechanism. First, the over-accumulated NCED in transgenic plants modified the pH in the cellular environment. Since the ABA signal in grapevine was shown to be intensified along with the pH gradient in vine (Li et al., 2011), we speculated the change of pH in plants expelled ABA from the synthesized location. Besides pH, the ABA accumulation is closely correlated with stomatal density, proline content (Yu et al., 2008), drought conditions, stomatal aperture and stomatal transpiration rate (reviewed by Kuromori et al., 2018). The difference of all these factors in each line of grapevines could have contributed to the difference of NCED gene expression level and ABA content in leaves.

Abscisic acid signaling pathway is a complex network, while JA share common targets in the signaling pathways of ABA (De Ollas and Dodd, 2016). In this study, the increase of ABA content in transgenic plants also induced accumulation of JA in grapevine leaves (**Figure 4F**). The expression level of *VvOPR3* is of same trend with ABA content under normal condition (**Figure 4E**). This is due to the fact that ABAregulates the induction of the *OPR3* gene in plants. This regulation was reported in a tomato mutant *flacca* (*flc*, ABA-deficient) (Muñoz-Espinoza et al., 2015). In this study, the level of *OPR3* remained above basal levels under drought condition, which suggested that JA and *OPR3* were regulated by ABA.

Salicylic acid exhibits synergistic effects with JA (Mur et al., 2006). However, in our study, the over-accumulation of ABA decreased SA content under normal condition (**Figure 4I**). This might be explained as the way ABA effect on SA is not rapidly but in a moderated pattern. This pattern is proved in *flc* (Muñoz-Espinoza et al., 2015). In their study, the SA level of *flc*s decreased in the first 3 h of drought treatment, then increased progressively for 24 h, which is different from the increased expression of ABA and JA level.

### Overexpression of *VaNCED1* Improved Growth Performance and Drought Resistance in Grapevine

Overexpression of *VaNCED1* increased the growth rate than WT. *NCED* gene has been transformed into many species, such as tomato (*S. lycopersicum*) (Thompson et al., 2000), *Arabidopsis thaliana* (Wan and Li, 2006), tobacco (*Nicotiana plumbaginifolia*) (Qin and Zeevaart, 2002), and tobacco (*Nicotiana tabacum*) (Zhang et al., 2008), resulting in the accumulation of ABA and improved drought resistance. While the *NCED*-silenced tomato mutant *not* (Thompson et al., 2004) greatly reduced shoot and root development than WT.

In the current study, although the growth rate of CVAN5 plants was higher than that of the WT and CVAN4 plants under slight drought stress, the ‘stunt’ CVAN4 plants showed the highest growth rate on day 6 (**Figure 5**). This might be explained by the dual roles of ABA in the physiological regulation of plant growth. A slight increase in ABA concentration (**Figure 4C**) could improve plant growth performance, whereas a great increase in ABA concentration could inhibit plant growth but help plants to survive through drought resistance.

### *VaNCED1* Decreased Stomatal Density and Photosynthesis in Grapevine

The improved drought resistance in transgenic plants might be induced by changes in stomata, photosynthesis system, antioxidants, and proline content. We first analyzed the stomatal density of leaf epithelial cells. Our results showed that stomatal density was significantly reduced in the transgenic CVAN4 plants, accompanied with excellent drought tolerance (**Figure 6**). Furthermore, the CVAN4 plants also showed decreased photosynthesis, which was consistent with the reduced stomatal density. It has been proved that the increased ABA content and decreased stomatal density were accompanied by improved drought tolerance in mutant or transgenic plants. One mutant screened from a drought–resistant pool of *Arabidopsis* showed reduced stomatal density accompanied by higher ABA and proline contents (Yu et al., 2008). Similarly, overexpression of a *Medicago truncatula* cold-acclimation specific protein 31 (MtCAS31) contributed to enhanced drought tolerance, higher ABA content, and reduced stomatal density (Xie et al., 2012). Reduced stomatal density is also known to affect water and CO_2_ exchange and to further affect the photosynthesis system in plants (Hetherington and Woodward, 2003).

### *VaNCED1* Decreased Proline and SOD Contents in Grapevine

Proline and SOD are two important factors that enable plants to cope with drought stress. Higher SOD activity and proline content contribute to better osmotic adjustment and ROS detoxification, which are important determinants of drought tolerance (Gupta et al., 1993; Kishor et al., 1995; De Ronde et al., 2004; Yu et al., 2008). In our study, the contents of proline and SOD were unexpectedly lower in the transgenic plants than in the WT plants under both normal and drought stress conditions (**Figure 7**). High levels of proline and SOD are generally believed to increase plant drought resistance; however, we suggested that proline and SOD contents are physiological indicators that reflect the extent of damage in plants, rather than drought resistance indices. The transgenic plants with better drought resistance exhibited lower levels of proline and SOD than those in the WT plants, indicating that CVAN4 and CVAN5 plants suffered less damage, compared to that in the WT after drought treatment. In contrast, the WT plants were more drought-sensitive, which led to high SOD and proline contents.

### *VaNCED1* Plays an Important Role in the Water Stress Transcriptome Network

*VvAREB1* and *VvABF2* are two important transcription factors in ABA signaling pathways (Giraudat et al., 1992). It was reported that under drought stress, *VvAREB1* and *VvABF2* genes were significantly upregulated in five grapevine genotypes under high (−1 MPa) and severe (−1.5 MPa) stress levels (Zandkarimi et al., 2015). In our study, compared to the WT plants, the expression levels of *VvAREB1*, *VvABF2*, and *VvPIP2* were higher in the resistant transgenic plants (**Figures 8B–D**). Furthermore, a higher accumulation of gene transcripts was observed under drought stress, compared to that under normal conditions, suggesting that *NCED* required a stress signal to trigger a more significant drought response. This implied the contribution of *NCED* to the enhanced stress tolerance in the transgenic plants.

Under normal and drought conditions, there was no difference in *VvCBF4* expression between transgenic plants and WT plants. However, the *VvCBF4* expression increased in both transgenic and WT plants after dehydration treatment (**Figure 8E**). These results implied that in grapevine, *VvCBF4* can be induced by drought stress, but probably through a ABA-independent way. Same result was reported in a *Arabidopsis* mutant’s *atx1* (Ding et al., 2011). The *CBF4* in *atx1* displayed ABA-independent and dehydration-inducible response. Zandkarimi et al. (2015) also proved *VvCBF4* can be induced by drought stress in all five grapevine varieties they tested.

The relationship between ABA level and expression level of *VvABI5* was proved varied depending on their drought resistant ability in grapevine (Hopper et al., 2016). The expression level of *VvABI5* in a drought tolerant genotype showed highly sensitive to dehydration, while in the sensitive genotype the *VvABI5* showed little respond to dehydration. *ABI5* also showed a compensation effect with *ABF1* and *ABF3* in *Arabidopsis* (Zandkarimi et al., 2015). Moreover, in our study, the expression level of *VvABI5* was highly induced by dehydration (**Figure 8F**), which is contrary to the expression pattern of *AtABI5* in *Arabidopsis*. The *AtABI5* in *Arabidopsis* is ABA-dependent, and can’t be induced by drought stress (Brocard et al., 2002). ABI was first cloned from ABA insensitive mutant of *Arabidopsis* (Finkelstein and Lynch, 2000). This gene family was then reported to be highly expressed in seed development (Fujita et al., 2011), seed dormancy (Miura et al., 2009; Shu et al., 2013, 2016a,b; Ding et al., 2014), and stomatal movement (Qiao et al., 2016). In *Arabidopsis*, the *ABI3* was reported collaboratively regulate seed dormancy with WRKY41 (Ding et al., 2014). *ABI4* was recently confirmed to play key role in ABA and GA antagonism in seed dormancy in *Arabidopsis* (Shu et al., 2013, 2016a,b). Moreover, a small ubiquitin-like modifier (SUMO) E3 ligase SIZ1 negatively regulates ABA signaling depending on *ABI5* in *Arabidopsis* (Miura et al., 2009). The SUMO T-DNA insertion mutations showed seed germination arrest and seedling primary root growth inhibition in seedlings.

## Conclusion

Cisgene transgenic technology is a powerful and clean tool for transferring genes between non-crossable species (Vanblaere et al., 2011). New transformation protocols allow genes to be inserted into plants without marker genes remaining inside the genome. The results of this study suggested that *VaNCED1* might be a promising candidate gene to improve drought tolerance in sensitive grapevine.

## Author Contributions

RH, YaZ, and JL designed the research. RH, YuZ, YC, CA, SL, JW, and SD performed the research. RH, YaZ, JL, MW, CA, and YuZ drafted the article.

## Conflict of Interest Statement

The authors declare that the research was conducted in the absence of any commercial or financial relationships that could be construed as a potential conflict of interest.

## Footnotes

1 http://www.genoscope.cns.fr/cgi-bin/blast_server/projet_ML/blast.pl

2 www.ncbi.nlm.nih.gov

## Supplementary Materials

FIGURE S1 Amino acid sequence alignment of the deduced amino acids of ZSYNCED with NCED from *Vitis vinifera* ‘Pinot Noir’ PNNCED1 (VV78X205727.5). Black color denotes the homologous regions.

FIGURE S2 Regenerated putative transgenic grapevine plants in normal **(A)** and malformed plants **(B)**. Arrows indicate malformed cotyledon and root.

## References

[B1] Agüero C. B. Meredith C. P. Dandekar A. M. (2006). Genetic transformation of *Vitis vinifera* L. cvs Thompson Seedless and Chardonnay with the pear PGIP and GFP encoding genes. *Vitis* 45 1–8. 10.1007/s11240-016-1023-4

[B2] Aroca R. Del Mar Alguacil M. Vernieri P. Ruiz-Lozano J. M. (2008). Plant responses to drought stress and exogenous ABA application are modulated differently by mycorrhization in tomato and an ABA-deficient mutant (sitiens). *Microb. Ecol.* 56 704–719. 10.1007/s00248-008-9390-y 18443845

[B3] Aroca R. Ferrante A. Vernieri P. Chrispeels M. J. (2006). Drought, abscisic acid and transpiration rate effects on the regulation of PIP aquaporin gene expression and abundance in *Phaseolus vulgaris* plants. *Ann. Bot.* 98 1301–1310. 10.1007/s10265-017-0920-x 17028296 PMC2803586

[B4] Ashraf M. Bashir A. (2003). Salt stress induced changes in some organic metabolites and ionic relations in nodules and other plant parts of two crop legumes differing in salt tolerance. *Flora* 198 486–498. 10.1078/0367-2530-00121

[B5] Aswath C. R. Kim S. H. Mo S. Y. Kim D. H. (2005). Transgenic plants of creeping bent grass harboring the stress inducible gene, 9-cis-epoxycarotenoid dioxygenase, are highly tolerant to drought and NaCl stress. *Plant Growth Regul.* 47 129–139. 10.5772/56096

[B6] Awan S. Z. Chandler J. O. Harrison P. J. Sergeant M. J. Bugg T. D. Thompson A. J. (2017). Promotion of germination using hydroxamic acid inhibitors of 9-cis-epoxycarotenoid dioxygenase. *Front. Plant Sci.* 8:357. 10.3389/fpls.2017.00357 28373878 PMC5357653

[B7] Bates L. S. Waldren R. P. Teare I. D. (1973). Rapid determination of free proline for water-stress studies. *Plant Soil* 39 205–207. 10.1007/BF00018060 20688380

[B8] Boneh U. Biton I. Schwartz A. Ben-Ari G. (2012). Characterization of the ABA signal transduction pathway in *Vitis vinifera*. *Plant Sci.* 187 89–96. 10.1016/j.plantsci.2012.01.015 22404836

[B9] Brocard I. M. Lynch T. J. Finkelstain R. R. (2002). Regulation and role of the arabidopsis abscisic acid-insensitive 5 gene in abscisic acid, sugar, and stress response. *Plant Physiol.* 129 1533–1543. 10.1104/pp.005793 12177466 PMC166741

[B10] Burbidge A. Grieve T. Jackson A. Thompson A. Taylor I. (1997). Structure and expression of a cDNA encoding a putative neoxanthin cleavage enzyme (NCE), isolated from a wilt-related tomato (*Lycopersicon esculentum* Mill.) library. *J. Exp. Bot.* 48 2111–2112. 10.1093/jxb/48.12.2111

[B11] Burbidge A. Grieve T. M. Jackson A. Thompson A. McCarty D. R. Taylor I. B. (1999). Characterization of the ABA-deficient tomato mutant notabilis and its relationship with maize Vp14. *Plant J.* 17 427–431. 10.1046/j.1365-313X.1999.00386.x 10205899

[B12] Chapman S. C. Chakraborty S. Dreccer M. F. Howden S. M. (2012). Plant adaptation to climate change-opportunities and priorities in breeding. *Crop Past. Sci.* 63 251–268. 10.1071/CP11303

[B13] Chen Z. Zheng Z. Huang J. Lai Z. Fan B. (2009). Biosynthesis of salicylic acid in plants. *Plant Signal. Behav.* 4 493–496. 10.4161/psb.4.6.8392 19816125 PMC2688294

[B14] Chernys J. T. Zeevaart J. A. (2000). Characterization of the 9-cis-epoxycarotenoid dioxygenase gene family and the regulation of abscisic acid biosynthesis in avocado. *Plant Physiol.* 124 343–354. 10.1104/pp.124.1.343 10982448 PMC59148

[B15] Cutler S. R. Rodriguez P. L. Finkelstein R. R. Abrams S. R. (2010). Abscisic acid: emergence of a core signaling network. *Annu. Rev. Plant Biol.* 61 651–679. 10.1146/annurev-arplant-042809-112122 20192755

[B16] De Ollas C. Dodd I. C. (2016). Physiological impacts of ABA-JA interactions under water-limitation. *Plant Mol. Biol.* 91 641–650. 10.1007/s11103-016-0503-6 27299601 PMC4932129

[B17] De Ronde J. A. Cress W. A. Krüger G. H. J. Strasser R. J. Van Staden J. (2004). Photosynthetic response of transgenic soybean plants, containing an *Arabidopsis P5CR* gene, during heat and drought stress. *J. Plant Physiol.* 161 1211–1224. 10.1016/j.jplph.2004.01.014 15602813

[B18] Ding Y. Avramova Z. Fromm M. (2011). The Arabidopsis trithorax-like factor ATX1 functions in dehydration stress responses via ABA-dependent and ABA-independent pathways. *Plant J.* 66 735–744. 10.1111/j.1365-313X.2011.04534.x 21309869

[B19] Ding Z. J. Yan J. Y. Li G. X. Wu Z. C. Zhang S. Q. Zheng S. J. (2014). WRKY41 controls Arabidopsis seed dormancy via direct regulation of ABI3 transcript levels not downstream of ABA. *Plant J.* 79 810–823. 10.1111/tpj.12597 24946881

[B20] Estrada-Melo A. C. Reid M. S. Jiang C. Z. (2015). Overexpression of an ABA biosynthesis gene using a stress-inducible promoter enhances drought resistance in petunia. *Hortic. Res.* 2:15013. 10.1038/hortres.2015.13 26504568 PMC4595983

[B21] Finkelstein R. R. Lynch T. J. (2000). The Arabidopsis abscisic acid response gene ABI5 encodes a basic leucine zipper transcription factor. *Plant Cell* 12 599–609. 10.1105/tpc.12.4.599 10760247 PMC139856

[B22] Franks T. Ding G. Thomas M. (1998). Regeneration of transgenic *Vitis vinifera* L. Sultana plants: genotypic and phenotypic analysis. *Mol. Breed.* 4 321–333. 10.1023/A:1009673619456

[B23] Fujita Y. Fujita M. Satoh R. Maruyama K. Parvez M. M. Seki M. (2005). AREB1 is a transcription activator of novel ABRE-dependent ABA signaling that enhances drought stress tolerance in Arabidopsis. *Plant Cell* 17 3470–3488. 10.1105/tpc.105.035659 16284313 PMC1315382

[B24] Fujita Y. Fujita M. Shinozaki K. Yamaguchi-Shinozaki K. (2011). ABA-mediated transcriptional regulation in response to osmotic stress in plants. *J. Plant Res.* 124 509–525. 10.1007/s10265-011-0412-3 21416314

[B25] Giraudat J. Hauge B. M. Valon C. Smalle J. Parcy F. Goodman H. M. (1992). Isolation of the Arabidopsis ABI3 gene by positional cloning. *Plant Cell* 4 1251–1261. 10.1105/tpc.4.10.1251 1359917 PMC160212

[B26] Gupta A. S. Webb R. P. Holaday A. S. Allen R. D. (1993). Overexpression of superoxide dismutase protects plants from oxidative stress (induction of ascorbate peroxidase in superoxide dismutase-overexpressing plants). *Plant Physiol.* 103 1067–1073. 10.1104/pp.103.4.1067 12232001 PMC159091

[B27] Hayat Q. Hayat S. Irfan M. Ahmad A. (2010). Effect of exogenous salicylic acid under changing environment: a review. *Environ. Exp. Bot.* 68 14–25. 10.1016/j.envexpbot.2009.08.005

[B28] He R. Wu J. Zhang Y. Agüero C. B. Li X. Liu S. (2017). Overexpression of a thaumatin-like protein gene from *Vitis amurensis* improves downy mildew resistance in *Vitis vinifera* grapevine. *Protoplasma* 254 1579–1589. 10.1007/s00709-016-1047-y 27900595

[B29] Hetherington A. M. Woodward F. I. (2003). The role of stomata in sensing and driving environmental change. *Nature* 424 901–908. 10.1038/nature01843 12931178

[B30] Hood E. E. Gelvin S. B. Melchers L. S. Hoekema A. (1993). New Agrobacterium helper plasmids for gene transfer to plants. *Transgenic Res.* 2 208–218. 10.1007/BF01977351

[B31] Hopper D. W. Ghan R. Schlauch K. A. Cramer G. R. (2016). Transcriptomic network analyses of leaf dehydration responses identify highly connected ABA and ethylene signaling hubs in three grapevine species differing in drought tolerance. *BMC Plant Biol.* 16:118. 10.1186/s12870-016-0804-6 27215785 PMC4877820

[B32] Hossain M. A. Cho J. I. Han M. Ahn C. H. Jeon J. S. An G. (2010). The ABRE-binding bZIP transcription factor OsABF2 is a positive regulator of abiotic stress and ABA signaling in rice. *J. Plant Physiol.* 167 1512–1520. 10.1007/s11103-009-9592-9 20576316

[B33] Iuchi S. Kobayashi M. Taji T. Naramoto M. Seki M. Kato T. (2001). Regulation of drought tolerance by gene manipulation of 9-cis-epoxycarotenoid dioxygenase, a key enzyme in abscisic acid biosynthesis in *Arabidopsis*. *Plant J.* 27 325–333. 10.1046/j.1365-313x.2001.01096.x 11532178

[B34] Iuchi S. Kobayashi M. Yamaguchi-Shinozaki K. Shinozaki K. (2000). A stress-inducible gene for 9-cis-epoxycarotenoid dioxygenase involved in abscisic acid biosynthesis under water stress in drought-tolerant cowpea. *Plant Physiol.* 123 553–562. 10.1104/pp.123.2.553 10859185 PMC59023

[B35] Jefferson R. A. (1987). Assaying chimeric genes in plants: the GUS gene fusion system. *Plant Mol. Biol. Rep.* 5 387–405. 10.1007/BF02667740

[B36] Kende H. Zeevaart J. (1997). The five” Classical” plant hormones. *Pant Cell* 9 1197–1210. 10.1105/tpc.9.7.1197 12237383 PMC156991

[B37] Kishor P. K. Hong Z. Miao G. H. Hu C. A. A. Verma D. P. S. (1995). Overexpression of Δ-pyrroline-5-carboxylate synthetase increases proline production and confers osmotolerance in transgenic plants. *Plant Physiol.* 108 1387–1394. 10.1104/pp.108.4.1387 12228549 PMC157516

[B38] Koornneef M. Jorna M. L. Brinkhorst-Van der Swan D. L. C. Karssen C. M. (1982). The isolation of abscisic acid (ABA) deficient mutants by selection of induced revertants in non-germinating gibberellin sensitive lines of *Arabidopsis thaliana* (L.) Heynh. *Theor. Appl. Genet.* 61 385–393. 10.1007/BF00272861 24270501

[B39] Koornneef M. Léon-Kloosterziel K. M. Schwartz S. H. Zeevaart J. A. (1998). The genetic and molecular dissection of abscisic acid biosynthesis and signal transduction in Arabidopsis. *Plant Physiol. Biochem.* 36 83–89. 10.1016/S0981-9428(98)80093-4

[B40] Koornneef M. Reuling G. Karssen C. M. (1984). The isolation and characterization of abscisic acid-insensitive mutants of *Arabidopsis thaliana*. *Physiol. Plant.* 61 377–383. 10.1111/j.1399-3054.1984.tb06343.x

[B41] Kuromori T. Seo M. Shinozaki K. (2018). ABA transport and plant water stress responses. *Trends Plant Sci.* 23 513–522. 10.1016/j.tplants.2018.04.001 29731225

[B42] Le Gall O. Torregrosa L. Danglot Y. Candresse T. Bouquet A. (1994). Agrobacterium-mediated genetic transformation of grapevine somatic embryos and regeneration of transgenic plants expressing the coat protein of grapevine chrome mosaic nepovirus (gcmv). *Plant Sci.* 102 161–170. 10.1016/0168-9452(94)90034-5

[B43] Lema-Rumińska J. Goncerzewicz K. Gabriel M. (2013). Influence of abscisic acid and sucrose on somatic embryogenesis in cactus *Copiapoa tenuissima* Ritt. forma *mostruosa*. *Sci. World J.* 2013:513985. 10.1155/2013/513985 23843737 PMC3694557

[B44] Li B. Feng Z. Xie M. Sun M. Zhao Y. Liang L. (2011). Modulation of the rootsourced ABA signal along its way to the shoot in *Vitis ripariax* x *Vitis labrusca* under water deficit. *J. Exp. Bot.* 62 1731–1741. 10.1093/jxb/erq390 21131549

[B45] Liu S. Wu J. Zhang P. Hasi G. Huang Y. Lu J. (2016). Response of phytohormones and correlation of SAR signal pathway genes to the different resistance levels of grapevine against *Plasmopara viticola* infection. *Plant Physiol. Biochem.* 107 56–66. 10.1016/j.plaphy.2016.05.020 27244101

[B46] Livak K. J. Schmittgen T. D. (2001). Analysis of relative gene expression data using real-time quantitative PCR and the 2^−ΔΔC_T_^ Method. *J. Methods* 25 402–408. 10.1006/meth.2001.1262 11846609

[B47] Lloyd G. McCown B. (1981). Commercially feasible micropropagation of Mountain laurel, Kalmia latifolia, by the use of shoot tip culture. *Proc Inter. Plant Prop. Soc.* 30 421–427.

[B48] Merkle S. A. Wiecko A. T. Sotak R. J. Sommer H. E. (1990). Maturation and conversion of *Liriodendron tulipifera* somatic embryos. *In vitro Cell. Dev. Biol.* 26 1086–1093. 10.1007/BF02623699

[B49] Miura K. Lee J. Jin J. B. Yoo C. Y. Miura T. Hasegawa P. M. (2009). Sumoylation of ABI5 by the Arabidopsis SUMO E3 ligase SIZ1 negatively regulates abscisic acid signaling. *Proc. Natl. Acad. Sci. U.S.A.* 106 5418–5423. 10.1073/pnas.0811088106 19276109 PMC2664011

[B50] Muñoz-Espinoza V. A. López-Climent M. F. Casaretto J. A. Gómez-Cadenas A. (2015). Water stress responses of tomato mutants impaired in hormone biosynthesis reveal abscisic acid, jasmonic acid and salicylic acid interactions. *Front. Plant Sci.* 6:997. 10.3389/fpls.2015.00997 26635826 PMC4649032

[B51] Mur L. A. J. Kenton P. Atzorn R. Miersch O. Wasternack C. (2006). The outcomes of concentration-specific interactions between salicylate and jasmonate signaling include synergy, antagonism, and oxidative stress leading to cell death. *Plant Physiol.* 140 249–262. 10.1104/pp.105.072348 16377744 PMC1326048

[B52] Murashige T. Skoog F. (1962). A revised medium for rapid growth and bio assays with tobacco tissue cultures. *Physiol. Plant* 15 473–479. 10.1111/j.1399-3054.1962.tb08052.x

[B53] Murray M. G. Thompson W. F. (1980). Rapid isolation of high molecular weight plant DNA. *Nucleic Acids Res.* 8 4321–4326. 10.1093/nar/8.19.4321 7433111 PMC324241

[B54] Nonogaki M. Sall K. Nambara E. Nonogaki H. (2014). Amplification of ABA biosynthesis and signaling through a positive feedback mechanism in seeds. *Plant J.* 78 527–539. 10.1111/tpj.12472 24520869

[B55] Parida A. K. Das A. B. Mohanty P. (2004). Defense potentials to NaCl in a mangrove, *Bruguiera parviflora*: differential changes of isoforms of some antioxidative enzymes. *J. Plant Physiol.* 161 531–542. 10.1078/0176-1617-01084 15202709

[B56] Pastenes C. Villalobos L. Rios N. Reyes F. Turgeon R. Franck N. (2014). Carbon partitioning to berries in water stressed grapevines: the role of active transport in leaves and fruits. *Environ. Exp. Bot.* 107 154–166. 10.1016/j.envexpbot.2014.06.009

[B57] Pedrosa A. M. Cidade L. C. Martins C. P. S. Macedo A. F. Neves D. M. Gomes F. P. (2017). Effect of overexpression of citrus 9-cis-epoxycarotenoid dioxygenase 3 (CsNCED3) on the physiological response to drought stress in transgenic tobacco. *Genet. Mol. Res.* 16:gmr16019292. 10.4238/gmr16019292 28362996

[B58] Qiao Z. Li C. L. Zhang W. (2016). WRKY1 regulates stomatal movement in drought-stressed *Arabidopsis thaliana*. *Plant Mol. Biol.* 91 53–65. 10.1007/s11103-016-0441-3 26820136

[B59] Qin X. Zeevaart J. A. (1999). The 9-cis-epoxycarotenoid cleavage reaction is the key regulatory step of abscisic acid biosynthesis in water-stressed bean. *Proc. Natl. Acad. Sci. U.S.A.* 96 15354–15361. 10.1073/pnas.96.26.15354 10611388 PMC24823

[B60] Qin X. Zeevaart J. A. (2002). Overexpression of a 9-cis-epoxycarotenoid dioxygenase gene in *Nicotiana plumbaginifolia* increases abscisic acid and phaseic acid levels and enhances drought tolerance. *Plant Physiol.* 128 544–551. 10.1104/pp.010663 11842158 PMC148917

[B61] Qu H. Deng J. (1994). Two cold resistant *Vitis amurensis* cultivars, ‘Zuoshan-1’ and ‘Zuoshan-2’. *Grape Cultiv. Wine Mak.* 1 20–21. 10.13414/j.cnki.zwpp.1994.01.010

[B62] Rodrigo M. J. Alquezar B. Zacarías L. (2006). Cloning and characterization of two 9-cis-epoxycarotenoid dioxygenase genes, differentially regulated during fruit maturation and under stress conditions, from orange (*Citrus sinensis* L. Osbeck). *J. Exp. Bot.* 57 633–643. 10.1093/jxb/erj048 16396998

[B63] Rodríguez-Gacio M. D. C. Matilla-Vázquez M. A. Matilla A. J. (2009). Seed dormancy and ABA signaling: the breakthrough goes on. *Plant Signal. Behav.* 4 1035–1048. 10.4161/psb.4.11.9902 19875942 PMC2819511

[B64] Schaller A. Stintzi A. (2009). Enzymes in jasmonate biosynthesis–structure, function, regulation. *Phytochemistry* 70 1532–1538. 10.1016/j.phytochem.2009.07.032 19703696

[B65] Schwartz S. H. Tan B. C. Gage D. A. Zeevaart J. A. McCarty D. R. (1997). Specific oxidative cleavage of carotenoids by VP14 of maize. *Science* 276 1872–1874. 10.1126/science.276.5320.1872 9188535

[B66] Seo M. Koshiba T. (2002). Complex regulation of ABA biosynthesis in plants. *Trends Plant Sci.* 7 41–48. 10.1016/S1360-1385(01)02187-2 11804826

[B67] Shu K. Chen Q. Wu Y. Liu R. Zhang H. Wang P. (2016a). ABI4 mediates antagonistic effects of abscisic acid and gibberellins at transcript and protein levels. *Plant J.* 85 348–361. 10.1111/tpj.13109 26708041

[B68] Shu K. Liu X. D. Xie Q. He Z. (2016b). Two faces of one seed: hormonal regulation of dormancy and germination. *Mol. Plant* 9 34–45. 10.1016/j.molp.2015.08.010 26343970

[B69] Shu K. Zhang H. Wang S. Chen M. Wu Y. Tang S. (2013). ABI4 regulates primary seed dormancy by regulating the biogenesis of abscisic acid and gibberellins in Arabidopsis. *PLoS Genet.* 9:e1003577. 10.1371/journal.pgen.1003577 23818868 PMC3688486

[B70] Soar C. J. Speirs J. Maffei S. M. Loveys B. R. (2004). Gradients in stomatal conductance, xylem sap ABA and bulk leaf ABA along canes of *Vitis vinifera* cv. Shiraz: molecular and physiological studies investigating their source. *Funct. Plant Biol.* 31 659–669. 10.1071/FP03238 32688937

[B71] Souza A. Batista V. G. Pinheiro M. P. Suassuna J. F. Lima L. M. D. Fernandes P. D. (2016). Expression of NCED gene in colored cotton genotypes subjected to water stress. *Rev. Bras. Eng. Agríc. Ambient.* 20 692–696. 10.1590/1807-1929/agriambi.v20n8p692-696

[B72] Sultana S. Tureèková V. Ho C. L. Napis S. Namasivayam P. (2014). Molecular cloning of a putative Acanthus ebracteatus-9-cis-epoxycarotenoid deoxygenase (AeNCED) and its overexpression in rice. *J. Crop Sci. Biotechnol.* 17 239–246. 10.1007/s12892-014-0006-4

[B73] Tan B. C. Schwartz S. H. Zeevaart J. A. McCarty D. R. (1997). Genetic control of abscisic acid biosynthesis in maize. *Proc. Natl. Acad. Sci. U.S.A.* 94 12235–12240. 10.1073/pnas.94.22.12235 9342392 PMC23760

[B74] Thompson A. J. Jackson A. C. Symonds R. C. Mulholland B. J. Dadswell A. R. Blake P. S. (2000). Ectopic expression of a tomato 9-cis-epoxycarotenoid dioxygenase gene causes over-production of abscisic acid. *Plant J.* 23 363–374. 10.1046/j.1365-313x.2000.00789.x 10929129

[B75] Thompson A. J. Thorne E. T. Burbidge A. Jackson A. C. Sharp R. E. Taylor I. B. (2004). Complementation of notabilis, an abscisic acid-deficient mutant of tomato: importance of sequence context and utility of partial complementation. *Plant Cell Environ.* 27 459–471. 10.1111/j.1365-3040.2003.01164.x

[B76] Tong S. M. Xi H. X. Ai K. J. Hou H. S. (2017). Overexpression of wheat TaNCED gene in Arabidopsis enhances tolerance to drought stress and delays seed germination. *Biol. Plant.* 61 64–72. 10.1007/s10535-016-0692-5

[B77] Tung S. A. Smeeton R. White C. A. Black C. R. Taylor I. B. Hilton H. W. (2008). Over-expression of LeNCED1 in tomato (*Solanum lycopersicum* L.) with the rbcS3C promoter allows recovery of lines that accumulate very high levels of abscisic acid and exhibit severe phenotypes. *Plant Cell Environ.* 31 968–981. 10.1111/j.1365-3040.2008.01812.x 18373621

[B78] Uno Y. Furihata T. Abe H. Yoshida R. Shinozaki K. Yamaguchi-Shinozaki K. (2000). Arabidopsis basic leucine zipper transcription factors involved in an abscisic acid-dependent signal transduction pathway under drought and high-salinity conditions. *Proc. Natl. Acad. Sci. U.S.A.* 97 11632–11637. 10.1073/pnas.190309197 11005831 PMC17252

[B79] Urano K. Maruyama K. Ogata Y. Morishita Y. Takeda M. Sakurai N. (2009). Characterization of the ABA-regulated global responses to dehydration in Arabidopsis by metabolomics. *Plant J.* 57 1065–1078. 10.1111/j.1365-313X.2008.03748.x 19036030

[B80] Van Camp W. Bowler C. Villarroel R. Tsang E. W. Van Montagu M. Inze D. (1990). Characterization of iron superoxide dismutase cDNAs from plants obtained by genetic complementation in *Escherichia coli*. *Proc. Natl. Acad. Sci. U.S.A.* 87 9903–9907. 10.1073/pnas.87.24.9903 2263641 PMC55282

[B81] Vanblaere T. Szankowski I. Schaart J. Schouten H. Flachowsky H. Broggini G. A. (2011). The development of a cisgenic apple plant. *J. Biotechnol.* 154 304–311. 10.1016/j.jbiotec.2011.05.013 21663775

[B82] Wan X. Li L. (2005). Molecular cloning and characterization of a dehydration-inducible cDNA encoding a putative 9-cis-epoxycarotenoid dioxygenase in *Arachis hygogaea* L. *DNA Seq.* 16 217–223. 10.1080/10425170500129785 16147878

[B83] Wan X. R. Li L. (2006). Regulation of ABA level and water-stress tolerance of Arabidopsis by ectopic expression of a peanut 9-cis-epoxycarotenoid dioxygenase gene. *Biochem. Biophys. Res. Commun.* 347 1030–1038. 10.1016/j.bbrc.2006.07.026 16870153

[B84] Xie C. Zhang R. Qu Y. Miao Z. Zhang Y. Shen X. (2012). Overexpression of MtCAS31 enhances drought tolerance in transgenic Arabidopsis by reducing stomatal density. *New Phytol.* 195 124–135. 10.1111/j.1469-8137.2012.04136.x 22510066

[B85] Yamada M. Morishita H. Urano K. Shiozaki N. Yamaguchi-Shinozaki K. Shinozaki K. (2005). Effects of free proline accumulation in petunias under drought stress. *J. Exp. Bot.* 56 1975–1981. 10.1093/jxb/eri195 15928013

[B86] Yu H. Chen X. Hong Y. Y. Wang Y. Xu P. Ke S. D. (2008). Activated expression of an Arabidopsis HD-START protein confers drought tolerance with improved root system and reduced stomatal density. *Plant Cell* 20 1134–1151. 10.1105/tpc.108.058263 18451323 PMC2390749

[B87] Zandkarimi H. Ebadi A. Salami S. A. Alizade H. Baisakh N. (2015). Analyzing the expression profile of AREB/ABF and DREB/CBF genes under drought and salinity stresses in grape (*Vitis vinifera* L.). *PLoS One* 10:e0134288. 10.1371/journal.pone.0134288 26230273 PMC4521911

[B88] Zhang Y. Yang J. Lu S. Cai J. Guo Z. (2008). Overexpressing SgNCED1 in tobacco increases ABA level, antioxidant enzyme activities, and stress tolerance. *J. Plant Growth Regul.* 27 151–158. 10.1007/s00344-008-9041-z

