# Supplementary figures and images for: Overexpression of 9-cis-Epoxycarotenoid Dioxygenase Cisgene in Grapevine Increases Drought Tolerance and Results in Pleiotropic Effects
Source: Front Plant Sci. 2018 Aug 3;9:970. doi: 10.3389/fpls.2018.00970 (PMC6085461; doi:10.3389/fpls.2018.00970)

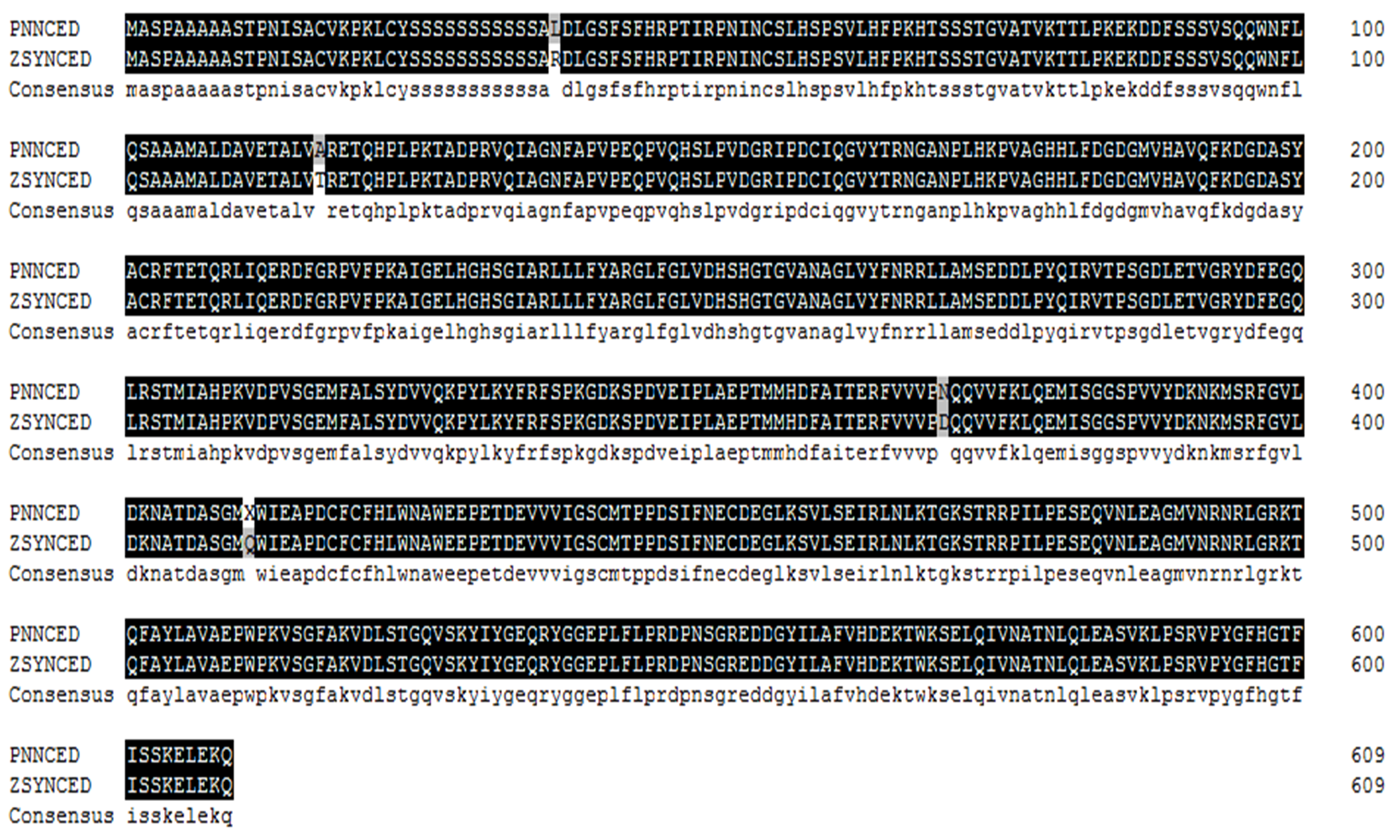

Supplement: FIGURE S1 — Amino acid sequence alignment of the deduced amino acids of ZSYNCED with NCED from Vitis vinifera ‘Pinot Noir’ PNNCED1 (VV78X205727.5). Black color denotes the homologous regions. [file Image_1.tif]

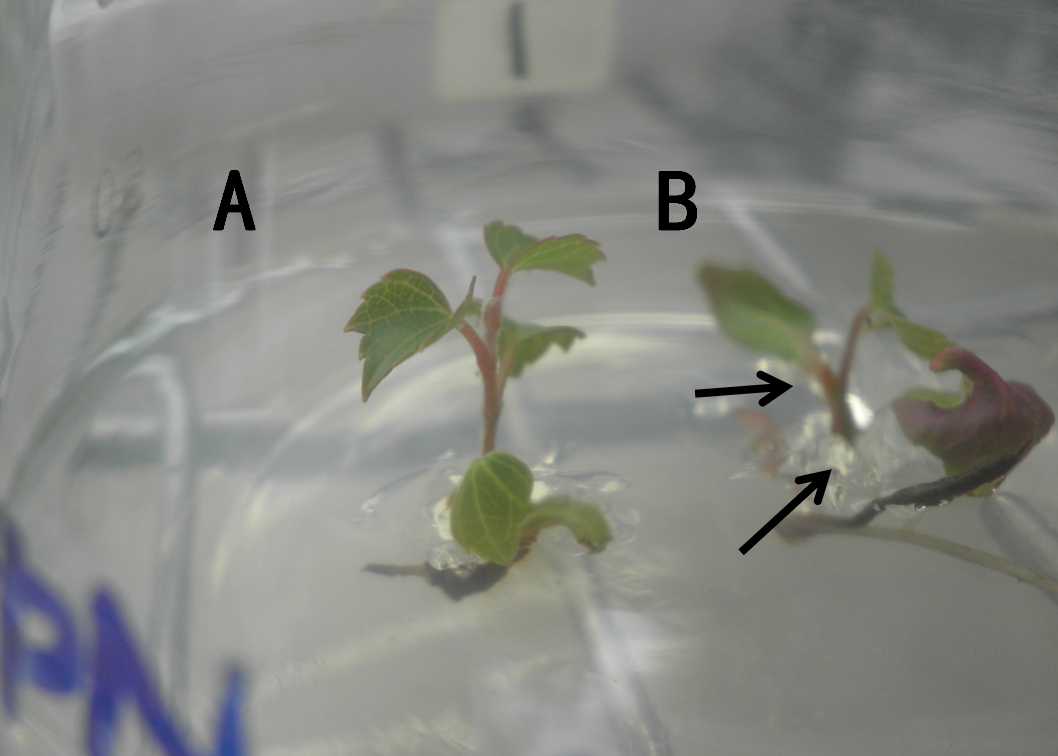

Supplement: FIGURE S2 — Regenerated putative transgenic grapevine plants in normal (A) and malformed plants (B). Arrows indicate malformed cotyledon and root. [file Image_2.tif]
